# How the Protein Environment Can Tune the Energy, the Coupling, and the Ultrafast Dynamics of Interacting Chlorophylls: The Example of the Water-Soluble Chlorophyll Protein

**DOI:** 10.1021/acs.jpclett.9b03628

**Published:** 2020-01-17

**Authors:** Elisa Fresch, Elena Meneghin, Alessandro Agostini, Harald Paulsen, Donatella Carbonera, Elisabetta Collini

**Affiliations:** † Department of Chemical Sciences, University of Padova, via Marzolo 1, 35131 Padua, Italy; ‡ Institute of Molecular Physiology, Johannes Gutenberg-University, Johannes-von-Müller-Weg 6, 55128 Mainz, Germany

## Abstract

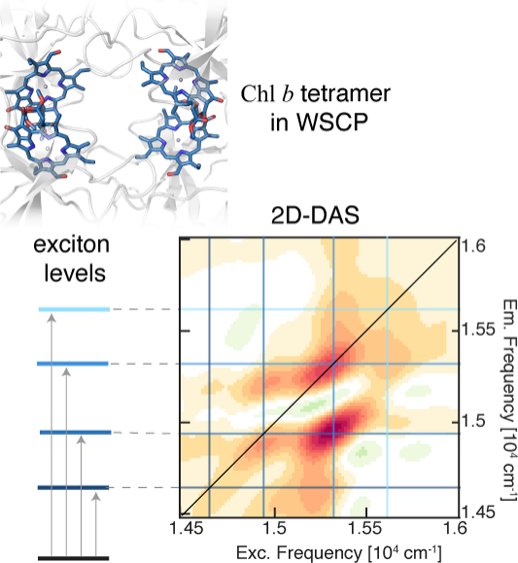

The interplay between active molecules and the protein environment in light-harvesting complexes tunes the photophysics and the dynamical properties of pigment–protein complexes in a subtle way, which is not fully understood. Here we characterized the photophysics and the ultrafast dynamics of four variants of the water-soluble chlorophyll protein (WSCP) as an ideal model system to study the behavior of strongly interacting chlorophylls. We found that when coordinated by the WSCP protein, the presence of the formyl group in chlorophyll *b* replacing the methyl group in chlorophyll *a* strongly affects the exciton energy and the dynamics of the system, opening up the possibility of tuning the photophysics and the transport properties of multichromophores by engineering specific interactions with the surroundings.

Biological systems, such as light-harvesting complexes, are characterized by optimized structures where the protein scaffold acts on the active molecules, finely tuning their surroundings and modulating their properties and functionalities. Numerous studies have addressed the contributions of individual amino acids to modulating the spectroscopic properties of bound chromophores, recognizing that they may act either by determining their 3D arrangement,^1−3^ thus affecting their interchromophore interactions, or by modifying their site energy.^3−17^ In several instances, these chromophore tunings have been achieved by means of hydrogen (H) bonds.^2,5−9,16,17^

Nevertheless, several details of the complex interplay between the active molecules and the protein environment at the molecular level are not fully clarified, and how to replicate the same mechanisms in artificial systems is still open to investigation.^18−20^ In this context, the water-soluble chlorophyll protein (WSCP) represents an ideal model system to investigate this kind of interaction more deeply.

WSCPs are a group of water-soluble proteins that contain only chlorophyll (Chl) molecules.^21^ Two classes of WSCPs are distinguished according to their photophysical properties: Class I WSCPs are subject to photoconversion,^22^ whereas class II WSCPs are not sensitive to illumination.^22−24^ Type II WSCPs can be further divided into classes IIa and IIb,^25^ distinguished by their different Chl-binding selectivities^26^ and the absorption spectra of the bound Chls.^15^ The structure of class II WSCPs, determined by X-ray crystallography, shows a tetrameric architecture formed by four identical subunits, each binding only one Chl molecule (Figure 1b).^15,27^ The four Chls are packed in a hydrophobic cavity in the protein matrix, forming two “open-sandwich”^28^ dimers (Figure 1).

**Figure 1:**
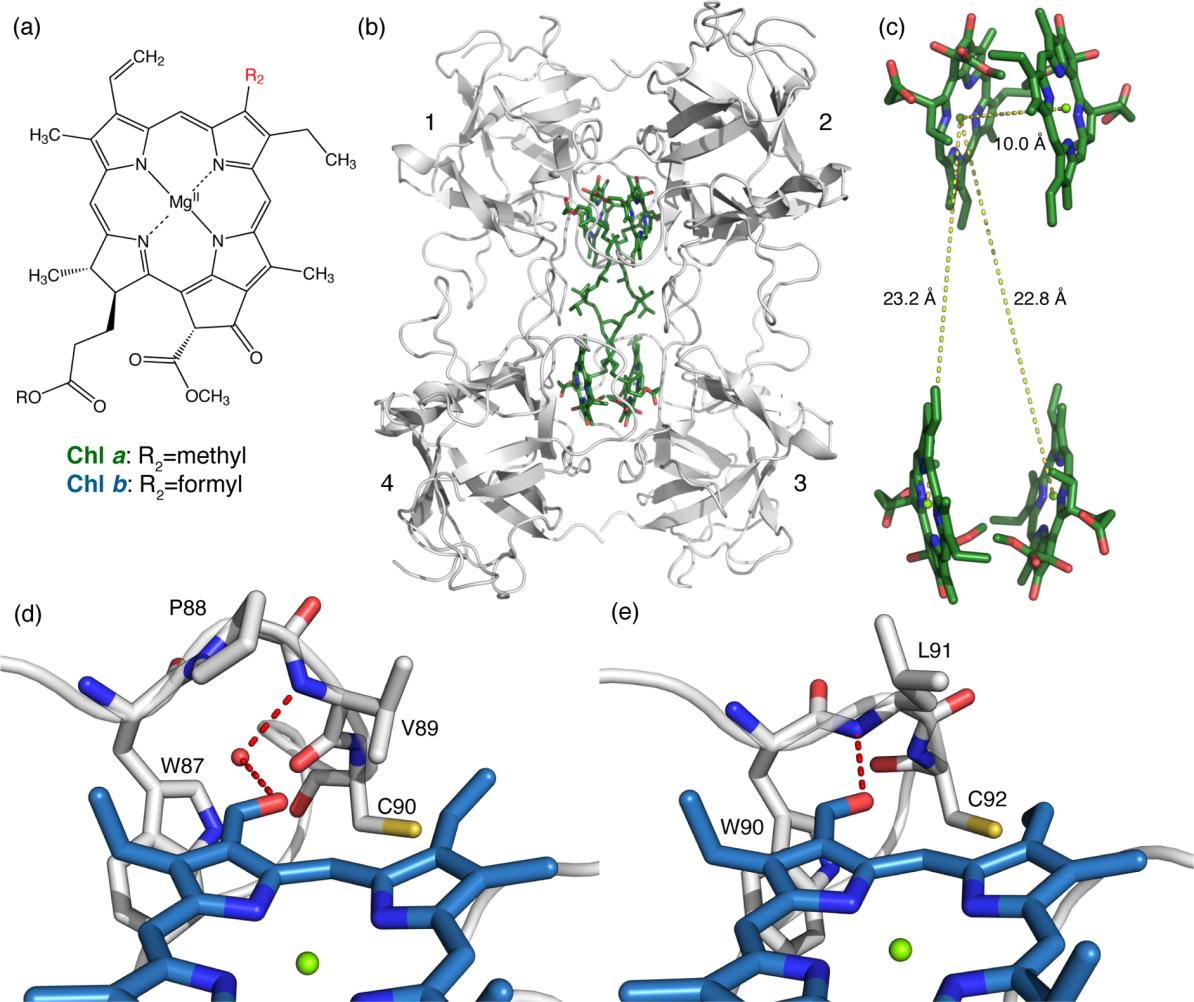
(a) Molecular structure of Chl *a* and Chl *b*. They differ in the R_2_ group in the C7 position. (b) Crystallographic structure of Bo-*a*,^15^ where the tetrameric architecture is recognizable. Chls *a* are shown as green sticks, and the protein scaffold is shown in gray. The structures of Bo-*b*, Lv-*a*, and Lv-*b* present similar tetrameric structures.^27,29^ (c) Focus on the arrangement of the Chl molecules inside the tetramer, organized in two “open-sandwich” dimers. Center–center distances are shown with yellow dashed lines. The phytyl chains are omitted for clarity. (d,e) Detail of the surroundings of Chl *b*’s formyl in (d) Bo-*b* and (e) Lv-*b*, pinpointing the H-bonds with red dashed lines. Chls *b* are shown as blue sticks, the protein is in gray. A red dot has been added to the position at which a water molecule is expected to be present in Bo-*b*.^29^

In this work the attention is focused on the ultrafast relaxation dynamics of four pigment–protein complexes, obtained by reconstituting two WSCPs, either from *Brassica oleracea* (belonging to class IIa, in the following denoted as Bo) or *Lepidium virginicum* (belonging to class IIb, denoted as Lv) with only Chl *a* or Chl *b* (Figure 1). The four resulting complexes are labeled Bo-*a*, Bo-*b*, Lv-*a*, and Lv-*b*, respectively. 2D electronic spectroscopy (2DES) is employed for this purpose, with the final goal of correlating the optical and dynamic response of the four complexes to the presence of specific pigment–protein interactions.

The absorption spectra of the four complexes at room temperature (RT, panels a and b) and 77 K (panel c) in the region of the Q bands are shown in Figure 2. In this spectral region, the protein spectra exhibit the typical features of Chl *a* and *b* chromophores, where Q_*y*_ and Q_*x*_ bands and their vibronic progressions are easily identified.^30−32^ The red shift of the Q_*y*_ maximum recorded for both Bo samples with respect to their Lv analogous has been attributed to a change in the site energy of the Chl molecules, induced by the deformation of the Chl macrocycle planarity in the Bo structure.^15^

**Figure 2:**
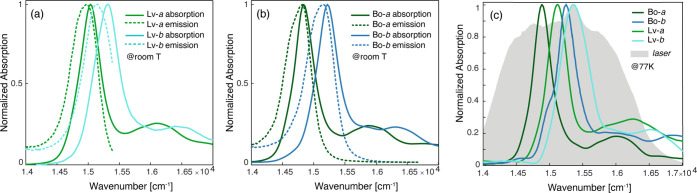
(a) Normalized absorption (solid line) and emission (dashed line) spectra of Lv-*a* (green) and Lv-*b* (light blue) in the Q-band region at room temperature. (b) Same as panel a for Bo-*a* (dark green) and Bo-*b* (dark blue). (c) Normalized absorption spectra at 77 K. The gray area represents the laser spectrum profile used in the 2DES experiments.

The spectra at 77 K are characterized by narrower and slightly blue-shifted peaks.^33^ Two main observations can be made by comparing the spectra of the four species. First, Lv complexes in both cases present a broader line shape; second, Chl *b*-binding proteins have a more resolved structure, with the appearance of clear shoulders on the red side of the spectra, which are less distinguishable in Chl *a* complexes. These features are attributed to excitonic states, as we will discuss later. The presence of excitonic interactions among Chls is confirmed by the CD spectra in the Vis region, where the typical behavior attributed to excitonic coupling among pigments can be observed in all of the samples (Figure S1 in the Supporting Information (SI)).

To evaluate the extent of the mutual Chl interactions in the samples, we calculated the value of the electronic coupling, *V*, expressed in terms of the dipole–dipole interaction, as detailed in the SI.^34^ In the calculation, the relative orientations of the transition dipole moments and the values of the interchromophore distances among the four chlorophylls have been obtained from the crystallographic structures,^15,27,29^ whereas the strength of the transition dipole moment of the pigments in the monomeric form has been approximated based on literature values (4.58 and 3.83 D for Chl *a* and *b*, respectively).^35^

These calculations led to the conclusion that in all proteins the four chlorophylls can be considered as forming two equivalent excitonic dimers (Chl 1–2 and 3–4, numbered as in Figure 1b), characterized by a dipole–dipole coupling *V* of 102, 67, 108, and 66 cm^–1^ for Lv-*a*, Lv-*b*, Bo-*a*, and Bo-*b*, respectively. The two dimers result in being weakly interacting as the mutual interactions between chlorophylls belonging to different dimers (Chl 1–3, 1–4, 2–3, and 2–4) are considerably lower (Table S9). This picture is in agreement with previous experimental works where the couplings among different pairs of chlorophylls were estimated by means of optically detected magnetic resonance.^36^

The calculated values for the strongly coupled dimers appear to be more dependent on the presence of Chl *a* or *b* than on the protein scaffolds. This reflects the similarity of the dimer arrangement in the X-ray structures of the four complexes.^15^

The values of *V* can be used for a first estimate of the energy gaps between excitonic states (calculated as 2*V*^34^), expected to be detected in the optical responses. On the basis of these calculations, the four excitonic states in the four complexes are expected to be almost two-by-two degenerate with an energy gap of ∼200 (130) cm^–1^ for Chl *a* (Chl *b*) proteins.

A better characterization of the electronic structure of the complexes can be obtained from 2DES measurements. 2DES is indeed one of the most powerful techniques for the determination of the excitonic energies and the quantification of electronic couplings thanks to its capability of spreading the optical response along two frequency dimensions in diagonal and off-diagonal coordinates.^37,38^

2DES measurements have been performed on the four samples at RT and 77 K. In all cases, the main feature in the 2D maps is a diagonal peak due to ground-state bleaching and stimulated emission involving the main electronic transitions addressed by the exciting profile. At RT, the broadening effects do not allow us to distinguish contributions from different excitonic states (Figure S4). At 77 K, instead, various features on and off the diagonal can be distinguished, as shown in Figure 3.

**Figure 3:**
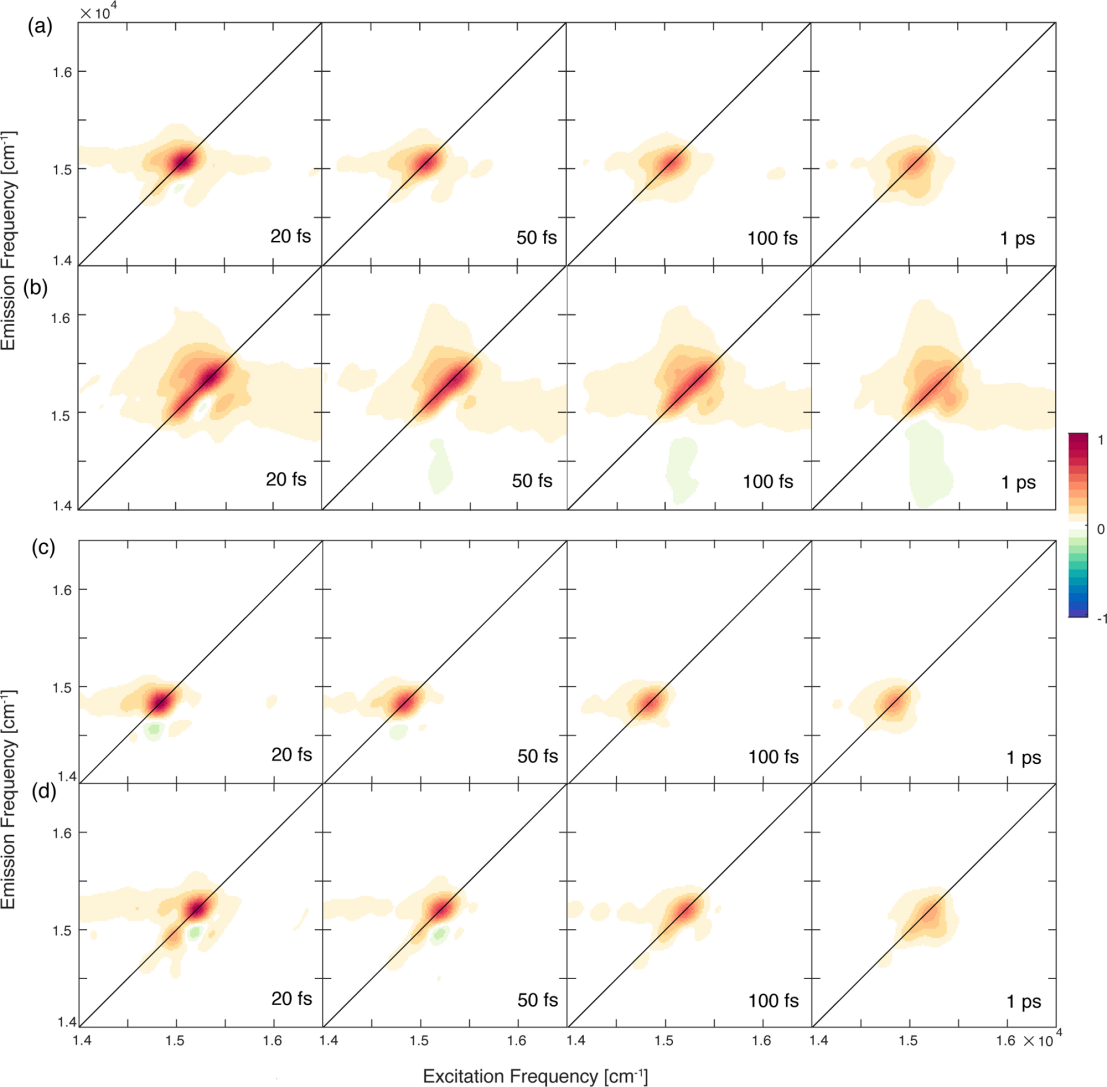
Evolution of 2DES maps at 77 K at selected values of population time *t*_2_ for (a) Lv-*a*, (b) Lv-*b*, (c) Bo-*a*, and (d) Bo-*b*.

From a first qualitative inspection of the maps, one can notice that the different bandwidth of the signals along the diagonal for the four samples follows the trend already noticed in the absorption spectra, with Lv samples and, in particular, Lv-*b* being characterized by the broadest bandwidth. Moreover, it is clear that both of the Chl *b*-reconstituted samples are characterized by a more complex signal distribution than their analogs binding Chl *a*. For example, comparing the 2D response of Bo-*a* and Bo-*b*, just two diagonal peaks at about 14 600 and 14 800 cm^–1^ can be identified in the former, whereas four signals can be pinpointed at about 14 620, 14 900, 15 300, and 15 650 cm^–1^ in the latter. Cross peaks across these features can also be ascertained, especially at early times.

To clarify the dynamics underlying the time evolution of the 2DES response at 77 K, the data have been analyzed through a global multiexponential fitting procedure,^39,40^ where the first 15 fs were omitted to minimize possible artifacts arising from pulse superposition.

The results of the fitting procedure are summarized in Figure 4 for Lv-*a* and Bo-*a* and in Figure 5 for Lv-*b* and Bo-*b*, where we report, for each time constant resulting from the fitting, the associated 2D-DAS (2D decay-associated spectrum). A 2D-DAS shows the amplitude distribution associated with a particular time constant from the fitting as a function of excitation and emission frequencies.^40^ A positive (negative) amplitude in a DAS means that the signal is exponentially decaying (rising) with the related time constant.

**Figure 4:**
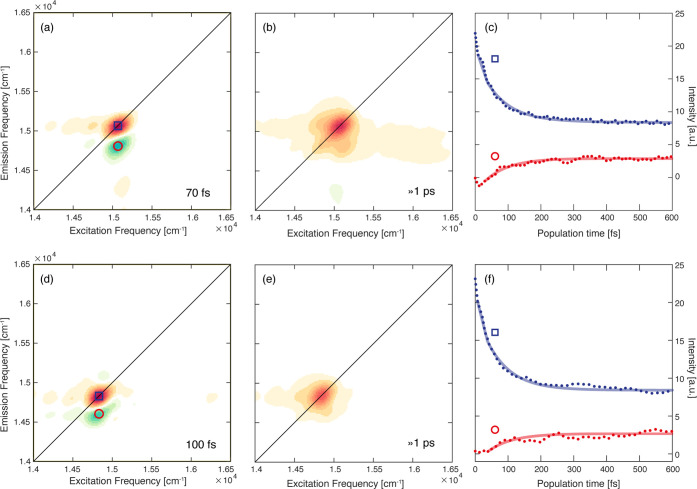
(a,b) 2D-DAS of Lv-*a* as obtained from the global fitting procedure of the 2DES data at 77 K. The associated time constants are reported in the panels. (c) Decay of the signal extracted at coordinates pinpointed by the blue square (15 070, 15 070) cm^*–*1^ and red circle (15 070, 14 850) cm^*–*1^. Dotted lines: experimental data; solid lines: fitting curves. (d–f) Same as before for Bo-*a*. The coordinates pinpointed by the blue square are (14 830, 14 830) cm^*–*1^, and those pinpointed by the red circle are (14 830, 14 600) cm^*–*1^.

**Figure 5:**
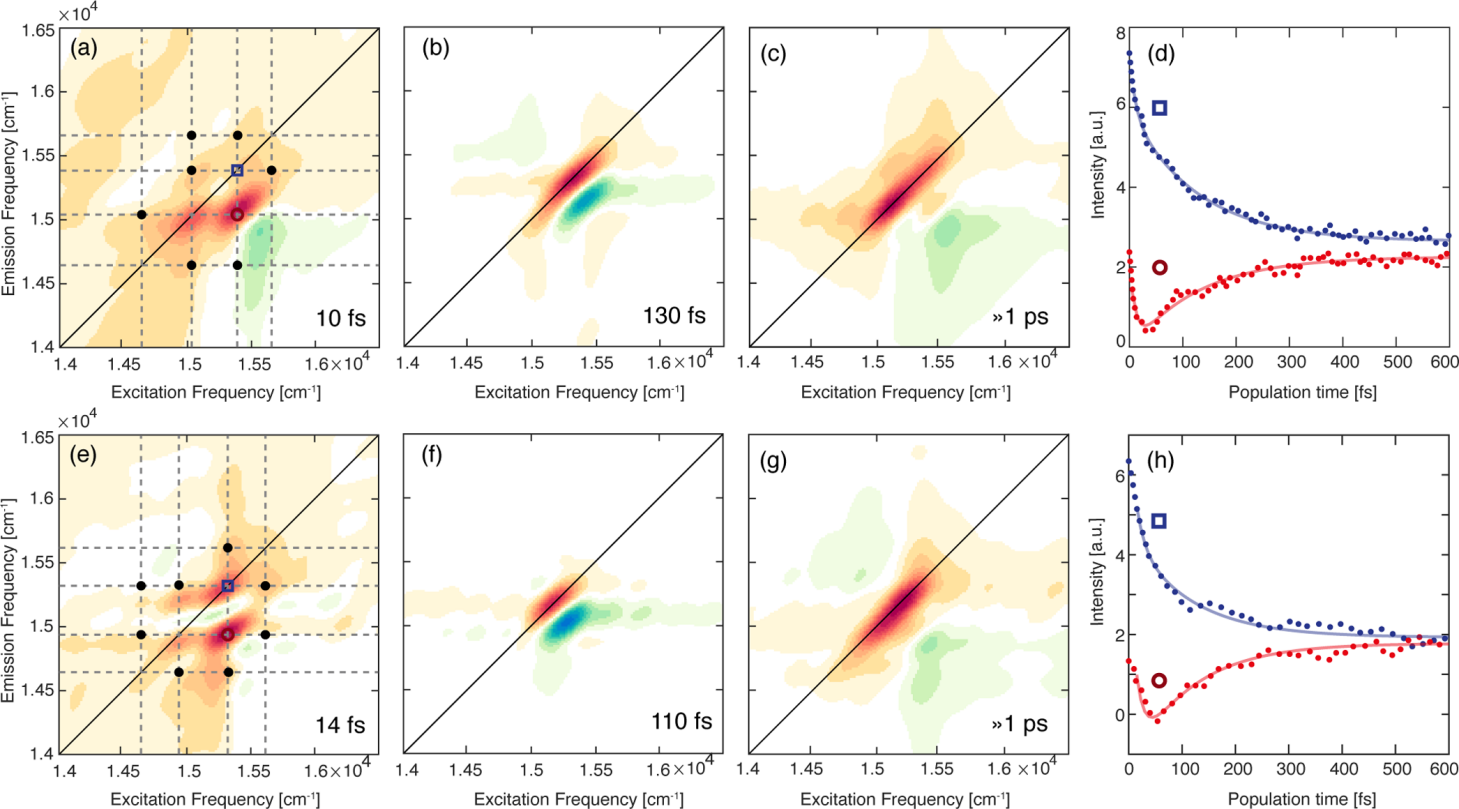
(a–c) 2D-DAS of Lv-*b* as obtained from the global fitting procedure of the 2DES data at 77 K. The associated time constants are reported in the panels. In panel a, the circles highlight the positions of the most intense cross peaks. (d) Decay of the signal extracted at coordinates pinpointed by the blue square (15 400, 15 400) cm^*–*1^ and red circle (15 400, 15 660) cm^*–*1^. Dotted lines: experimental data; solid lines: fitting curve. (e–h) Same as panels a–d for Bo-*b*. The coordinates pinpointed by the blue square are (15 300, 15 300) cm^*–*1^, and those pinpointed by the red circle are (15 300, 14 900) cm^*–*1^.

For Lv-*a* and Bo-*a*, the global fitting analysis revealed very similar kinetics, described by a biexponential function with two time constants, as can also be deduced from the decay traces extracted at selected coordinates (Figure 4c,f). In both samples, the first component has a characteristic time of ∼100 fs. The associated amplitude distributions (Figure 4a,d for Lv-*a* and Bo-*a*, respectively) present a positive amplitude signal in correspondence with the diagonal peak and a negative signal below the diagonal, typically attributed to relaxation processes from higher to lower energy states.^41−43^ The assignment of this kinetic constant to exciton relaxation is also in agreement with the calculated lifetime of 50–80 fs reported in the literature for excitons in homodimers of Chl *a* in the WSCP^44^ and with previous experiments on the wild-type class IIb WSCP.^33^ The coordinates of the positive and negative signals in the 2D-DAS allow us to estimate the excitonic energy gap between the initial and the final states. In both proteins, this gap is ∼220 cm^–1^, which is in good agreement with the estimate of the coupling *V* based on the dipole–dipole interaction and on the geometrical assembly of the chromophores described in the previous section (Δ*E* = 2*V*, ∼204 and ∼216 cm^–1^ for Lv-*a* and Bo-*a*, respectively).

The second time constant (>1 ps) (Figure 4b,e) accounts for all of the relaxation dynamics characterized by time scales longer than the investigated time window.

The behavior of the samples binding Chl *b* is different, as summarized in Figure 5. In this case, three components were necessary to reasonably fit the experimental time behavior in both cases. In addition to two time constants very similar to those for Lv-*a* and Bo-*a* (one on the order of 100 fs and another one >1 ps), a third ultrafast component on the order of ∼10 fs was found. The need for at least a three-exponential fitting model is also clearly recognizable in the representative decay traces extracted from the diagonal and off-diagonal coordinates, as shown in Figure 5d,h, where the presence of an additional ultrafast component is particularly evident, especially at cross-peaks coordinates (red traces). The validity of this ultrafast component is also proved by its peculiar amplitude distribution across the 2D maps, as verified through the associated 2D-DAS.

The 2D-DAS relative to the first two components (Figure 5b,f and c,g) are very similar to the corresponding ones found for Lv-*a* and Bo*-a*, suggesting the same origin of the associated dynamic phenomena. However, the broader distribution of the signals in these DAS already indicates that in Lv-*b* and Bo-*b*, the excitonic states involved in the relaxation dynamics are more spread in energy than those in Lv-*a* and Bo-*a*.

The 2D-DAS of the shortest time constant exhibit several features in both samples. In the case of Lv-*b*, signals at diagonal and cross-peak positions between coordinates 14 660, 15 050, 15 400, and 15 660 cm^–1^ can be identified, as pinpointed by the gray lines in Figure 5a. The positive amplitude at these coordinates means that the signal decays with a characteristic time of 10 fs. Similarly, in Bo-*b*, diagonal and cross-peak positions between coordinates 14 620, 14 900, 15 300, and 15 650 cm^–1^ are identified (Figure 5e).

These dynamics are attributed to the ultrafast dephasing of the coherent superpositions of excitonic states instantaneously prepared by the exciting pulses. These superpositions immediately dephase after photoexcitation; therefore, we cannot attribute any functional meaning to these overdamped coherences. Notwithstanding, the amplitude distribution of the signal is particularly helpful for pinpointing with greater precision the energies of the excitonic states in the early stages of the relaxation dynamics.^38^ The coordinates of the signals appearing in the 2D-DAS of Figure 5a,e can thus be used as an estimate of the energy of the excitonic states. In both Chl *b* complexes, four excitonic states can be reasonably identified. The energy of diagonal features is in agreement with the position of the weak shoulders previously identified on the red side of the absorption spectra at 77 K (Figure 2c). Moreover, the presence of signals at cross-peak positions between the main diagonal peaks at symmetric positions with respect to the diagonal (pinpointed in Figures 5a,e with black dots) and appearing immediately after photoexcitation is the typical manifestation of excitonic coupling.^37,38^

The energy separations among the excitonic states in both Chl *b* samples are significantly higher than the energy gaps estimated from the calculation of the dipole–dipole coupling *V* (Δ*E* ≈ 134 cm^–1^ for both Lv-*b* and Bo-*b*; see Table S9) and also higher than the ones measured in Chl *a* samples. They are also greater than the values previously determined through fluorescence line narrowing (FLN) and hole burning (HB) spectroscopy.^45−47^ This is not unreasonable, especially considering the different origins and time scales of the 2DES and HB signals: Whereas the HB spectra are dominated by the slow time scale energy evolution of the system, 2DES (and more in general, photon echo experiments) is performed with a broadband laser pulse that is able to simultaneously excite all of the transition frequencies and to promote the formation of coherent superpositions of different states.^48^ In this sense, 2DES provides an instantaneous picture of the excitonic levels, all simultaneously addressed, before any relaxation phenomenon takes place.^38^

The presence of four distinguishable signals suggests that the four Chl *b* molecules in Lv-*b* and Bo-*b* cannot be simply considered as a pair of dimers; rather, they constitute a tetramer with non-negligible couplings among all four pigments. This was also suggested by recent optically detected magnetic resonance experiments.^36^ On the contrary, assuming that the X-ray structure is providing the correct angles and distances, such an underestimated *V* coupling may only be derived from considering a wrong initial value of the Chl *b* transition dipole moment. This value, estimated to be >7 D, clearly does not correspond to the transition dipole moment of the isolated Chl *b* pigment in solution. Surprisingly, this discrepancy was observed only in Chl *b*-containing samples, unlike the case of Chl *a*.

This peculiar property of Chl *b* can be related to a parallel result that has already been noticed by analyzing the extinction coefficients of Chls *a* and *b* upon binding to the WSCP. The extinction coefficients of Chls *a* and *b* have a comparable dependence on the polarity of the surroundings. (See the SI.) However, Palm et al.^26^ noticed that Chl *b* embedded in the wild-type WSCP from either *B. oleracea* or *L. virginicum* exhibits an unexpectedly high molar extinction coefficient when compared with the corresponding Chl *a*-reconstituted complexes. Because of the well-known quadratic relation between the transition dipole moment and the molar extinction coefficient, this finding seems to point to a particularly large transition dipole moment of Chl *b* upon its binding inside the WSCP, in agreement with the results presented in this work.

Now, the intriguing point is to understand which structural factors may cause the different behaviors manifested by Chl *a* and Chl *b* in the same protein scaffold. The obvious starting point in this discussion is the only structural difference between the two pigments, that is, the presence of the formyl group in Chl *b* at the C7 position. It is known that the formyl group modifies the optical properties of the chromophore^49^ and can significantly contribute to the binding of Chl *b* to proteins, also thanks to the formation of H-bond networks.^24,26,29,50−52^ Carbonyl groups can also modify the charge distribution and thus the transition dipole moment^53−55^ through polarization effects or the formation of H-bonds. These two mechanisms are typically correlated and not easily distinguishable in complex media;^56^ however, the mere polarization effect can be excluded considering that, as previously discussed, the extinction coefficient of Chl *b* depends on the solvent’s polarity in a manner comparable to Chl *a*. Even in solvents able to establish H-bonds with the formyl group, like methanol, the effect is not present.

The most likely conclusion is that the electronic properties of the pigments (the transition dipole moment and then, in turn, also the electronic coupling and the excitonic energy gaps) are tuned by the presence of specific and directional interactions between the protein backbone and the formyl group on the Chl *b* moiety, mainly identified as H-bonds with specific amino acids, with or without the mediating action of a water molecule (Figure 1d,e).^29^ Interestingly, a similar argument has also been invoked to explain the different behaviors of Bo-*a* and Bo-*b* in HB experiments.^47^

This implies a particularly interesting and not yet fully explored role of the scaffold in pigment–protein complexes, where the effects are not limited to variations of the site energies of the pigments. Still, significant changes in the electronic coupling, energy gaps, and ultrafast time constants can be promoted through the establishment of specific and directional interactions, such as H-bonds. Further investigations, also through the 2DES characterization of WSCP variants, are needed to fully characterize the mechanism and to determine if specific H-bonds are the main cause of the observed enhancement of the dipole moment in bound Chl *b*.

In conclusion, we have demonstrated that the effect of the protein scaffold in pigment–protein complexes is not limited to variations of the site energies of the pigments, as largely documented in the literature,^4,7−9,11,15−17^ but that the establishment of specific and directional interactions can have very strong consequences for the electronic coupling and for the ultrafast dynamics of pigment–protein complexes as well. This is a particularly important finding because beyond having characterized this behavior in the WSCP, it implies the possibility of tuning the photophysics and the transport properties of multichromophores by engineering specific interactions with the surroundings. With respect to other supramolecular interactions, H-bonds appear to be particularly suited for this control task because of the possibility of more easily predictable orientations, distances, and geometries. In this context, our findings allow a step forward toward further investigations aimed to more consciously exploit these important interactions.

## Experimental Methods

WSCP complexes were expressed and reconstituted with purified Chls according to the protocol described by Agostini et al.^23^ and diluted in sodium phosphate buffer (20 mM, pH 7.8). Possible contaminations due to the presence of residual Chl *a* molecules in Bo-*b* and Lv-*b* have been excluded based on 77 K fluorescence measurements. For the 2DES experiments at 77 K, the samples were mixed with 60% glycerol (v/v) until an optical density of ∼0.3 in a 0.5 mm cuvette was reached on the Q_*y*_ maximum. For each sample, steady-state absorption spectra were acquired before and after each scan to control that no degradation of the sample had happened during the 2DES measurements.

2DES measurements were performed in the fully noncollinear BOXCARS (photon echo) geometry using the setup described in ref (57). The laser spectrum was centered at 15 380 cm^–1^ (650 nm) to cover the Q_*y*_ spectral region, as shown in Figure 2c. The pulse duration, optimized through FROG measures, was compressed to 8 fs, corresponding to a spectral bandwidth of ∼1840 cm^–1^ (Figure S3). The population time (*t*_2_) was scanned from 0 to 1000 fs, in steps of 7.5 fs, while the coherence time (*t*_1_) was scanned from 0 to 125 fs in steps of 3 fs. The exciting energy on the samples was ∼7 nJ per pulse. The measurements have been performed under the same conditions at RT and at 77 K, employing an Oxford Instruments OptistatDN cryostat. Each experiment was repeated three times to ensure reproducibility. The data analysis was performed by exploiting the global fitting methodology described in ref (40).

## Supporting Information Available

Additional linear characterization and 2DES data, 2DES pulse characterization, and calculation of the electronic coupling (PDF)
