# Supplementary material for: How the Protein Environment Can Tune the Energy, the Coupling, and the Ultrafast Dynamics of Interacting Chlorophylls: The Example of the Water-Soluble Chlorophyll Protein
Source: J Phys Chem Lett. 2020 Jan 17;11(3):1059–67. doi: 10.1021/acs.jpclett.9b03628 (PMC7995254; doi:10.1021/acs.jpclett.9b03628)
Supplement: Supplementary file 1 — jz9b03628_si_001.pdf [file jz9b03628_si_001.pdf]

SUPPORTING INFO

How the Protein Environment Can Tune the Energy, the Coupling and the Ultrafast Dynamics of Interacting Chlorophylls: the Example of the Water-Soluble Chlorophyll Protein

Elisa Fresch[1], Elena Meneghin[1], Alessandro Agostini[1,2], Harald Paulsen[2], Donatella Carbonera[1], and Elisabetta Collini[1,*]

[1]Department of Chemical Sciences, University of Padova, via Marzolo 1, 35131, Padova, Italy.

[2]Institute of Molecular Physiology, Johannes Gutenberg-University, Johannes-von-Müller-Weg 6, 55128 Mainz, Germany

* email: elisabetta.collini@unipd.it

Contents

S1. Calculation of the electronic coupling *V*

S2. Linear properties

S3. 2DES pulse characterization

S4. Additional 2DES data

S1. Calculation of the electronic coupling *V*

The electronic coupling V is expressed in terms of dipole-dipole interaction: [1]

$$V_{mn} = \frac{f_1^2}{4\pi\varepsilon_0\varepsilon_r}\left[\frac{\boldsymbol{\mu}_\alpha^m \cdot \boldsymbol{\mu}_\alpha^n}{\boldsymbol{r}_{mn}^3} - 3\frac{(\boldsymbol{\mu}_\alpha^m \cdot \boldsymbol{r}_{mn})(\boldsymbol{\mu}_\alpha^n \cdot \boldsymbol{r}_{mn})}{\boldsymbol{r}_{mn}^5}\right]$$

where the subscripts m and n run over the four Chls molecules in the WSCPs, $\boldsymbol{\mu}_\alpha^m$ is the transition dipole moment of the isolated chromophores in a spherical cavity (4.58 for Chl *a* and 3.83 Debye for Chl *b*) [2] and $\boldsymbol{r}_{mn}$ is the relative distance between pairs of chromophores. ε_0 is the dielectric constant in the vacuum, ε_r is the relative dielectric constant taken to be 2.40, as derived from a refractive index of 1.55 typical for protein environments[3]; f_1^2 is the local-field correction, considering the molecules embedded in a spherical cavity and surrounded by a dielectric medium:

$$f_1^2 = \frac{\varepsilon_r + 2}{3}$$

The relative orientations of the transition dipole moments and the values of the inter-chromophore distances have been obtained from the crystallographic structure and are summarized in Tables S1-S8. The calculated *V* among pairs of Chls for the four WSCPs are reported in Table S9. In the Tables the four Chls are numbered as in Figure 1(b) of the main text.

Geometrical parameters extracted from the X-ray structures for Lv-a.

Table S1. Inter-chromophore distances in Å obtained from the crystallographic structure for Lv-*a*.

Lv-a	*Chl-1*	*Chl-2*	*Chl-3*	*Chl-4*
Chl-1	\	9.94	21.2	19.4
Chl-2	9.94	\	19.2	20.9
Chl-3	21.2	19.3	\	9.98
Chl-4	19.3	20.9	9.98	\

Table S2. Relative orientations in Debye of the transition dipole moments for Lv-*a*.

Lv-a	*x*	*y*	*z*
Chl-1	1.20	-1.63	4.1
Chl-2	3.09	-1.89	2.78
Chl-3	-1.20	2.47	-3.66
Chl-4	-3.09	1.11	-3.20

Geometrical parameters extracted from the X-ray structures for Lv-b.

Table S3. Inter-chromophore distances in Å obtained from the crystallographic structure for Lv-*b*.

Lv-b	*Chl-1*	*Chl-2*	*Chl-3*	*Chl-4*
Chl-1	\	10.1	21.5	19.5
Chl-2	10.1	\	1.9539e-09	21.3
Chl-3	21.5	19.5	\	10.1
Chl-4	19.5	21.3	10.1	\

Table S4. Relative orientations in Debye of the transition dipole moments for Lv-*b*.

Lv-b	*x*	*y*	*z*
Chl-1	1.08	-1.26	3.45
Chl-2	2.58	-1.60	2.34
Chl-3	-1.02	2.13	-3.01
Chl-4	-2.52	0.89	-2.74

Geometrical parameters extracted from the X-ray structures for Bo-a.

Table S5. Inter-chromophore distances in Å obtained from the crystallographic structure for Bo-*a*.

Bo-a	*Chl-1*	*Chl-2*	*Chl-3*	*Chl-4*
Chl-1	\	9.79	23.1	22.8
Chl-2	9.79	\	2.2847e-09	23.1
Chl-3	23.1	22.8	\	9.79
Chl-4	22.8	23.1	9.79	\

Table S6. Relative orientations in Debye of the transition dipole moments for Bo-*a*.

Bo-a	*x*	*y*	*z*
Chl-1	-1.01	-3.89	-2.20
Chl-2	0.92	-2.87	-3.45
Chl-3	0.92	2.87	3.45
Chl-4	-1.01	3.89	2.20

Geometrical parameters extracted from the X-ray structures for Bo-b.

Table S7. Inter-chromophore distances in Å obtained from the crystallographic structure for Bo-*b*.

Bo-b	*Chl-1*	*Chl-2*	*Chl-3*	*Chl-4*
Chl-1	\	10.1	23.9	23.4
Chl-2	10.1	\	23.4	23.9
Chl-3	23.9	23.4	\	10.1
Chl-4	23.4	23.9	10.1	\

Table S8. Relative orientations in Debye of the transition dipole moments for Bo-*b*.

Bo-b	*x*	*y*	*z*
Chl-1	-3.75	0.47	-0.59
Chl-2	-3.75	-0.47	0.59
Chl-3	3.75	0.47	0.59
Chl-4	3.75	-0.47	-0.59

Table S.9. Calculated values of dipole-dipole interaction V in cm^{-1} for each pair of Chls (numbered as in Figure 1(b) of the main text) in the four WSCPs.

Lv-a	*Chl-1*	*Chl-2*	*Chl-3*	*Chl-4*
Chl-1	\	102.60	7.26	22.46
Chl-2	102.60	\	22.45	8.48
Chl-3	7.26	22.45	\	101.58
Chl-4	22.46	8.48	101.58	\

Lv-b	*Chl-1*	*Chl-2*	*Chl-3*	*Chl-4*
Chl-1	\	67.51	5.21	15.02
Chl-2	67.51	\	14.86	5.68
Chl-3	5.21	14.86	\	66.60
Chl-4	15.02	5.68	66.60	\

Bo-a	*Chl-1*	*Chl-2*	*Chl-3*	*Chl-4*
Chl-1	\	108.31	9.12	10.89
Chl-2	108.31	\	11.23	9.12
Chl-3	9.12	10.89	\	108.31
Chl-4	11.22	9.12	108.31	\

Bo-b	*Chl-1*	*Chl-2*	*Chl-3*	*Chl-4*
Chl-1	\	66.63	6.37	7.96
Chl-2	66.63	\	7.96	6.37
Chl-3	6.37	7.96	\	66.63
Chl-4	7.96	6.37	66.63	\

S2. Linear Properties

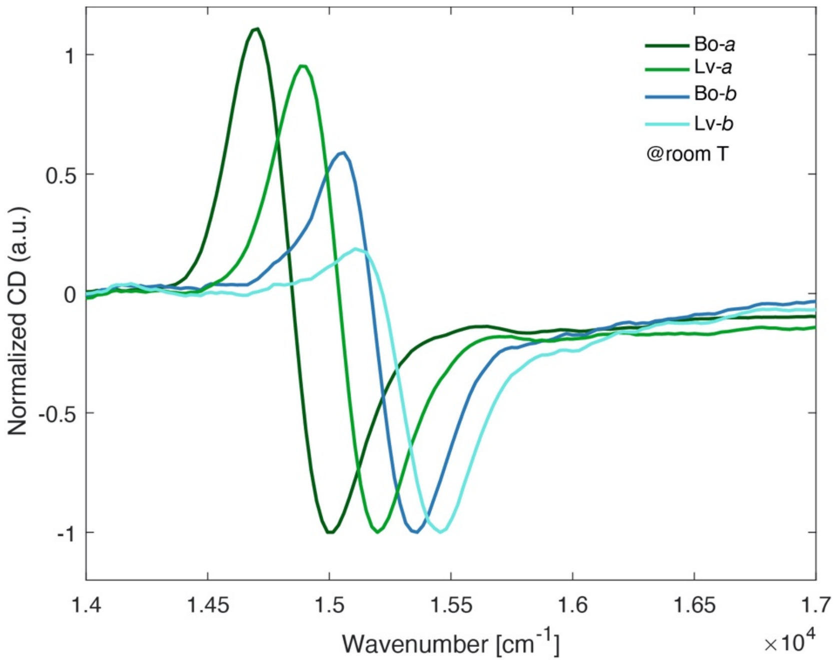

Figure S1. CD spectra of the four complexes at room temperature normalized on the negative peak. The spectra of the Chl *b*-complexes, especially Lv-*b* are non-conservative, in contrast to those of the Chl *a* complexes, indicating higher-order couplings.[4]

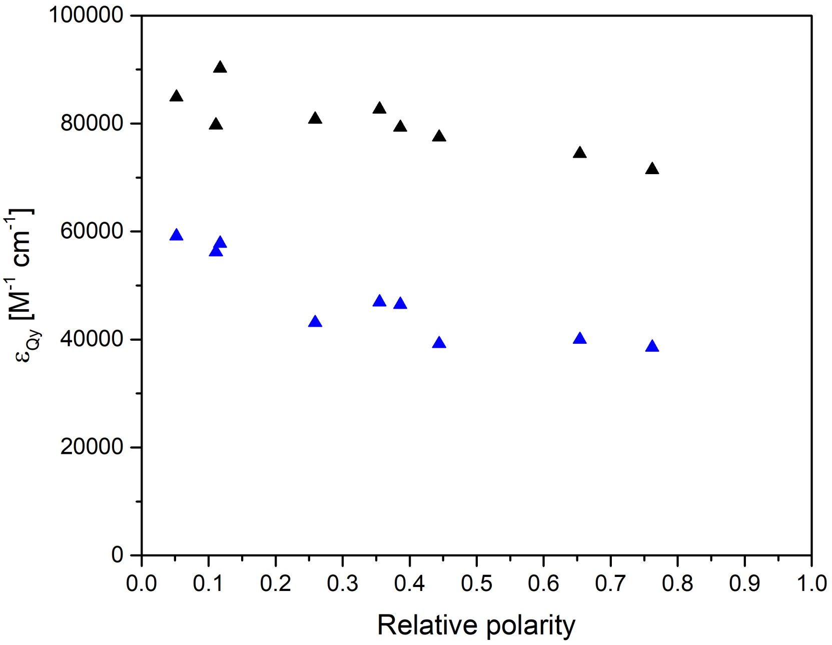

Figure S2. Molar extinction coefficient at Q_y maximum wavelength for Chl *a* (black triangles) and Chl *b* (blue triangles) in various solvents: carbon tetrachloride,[5] benzene,[6] diethyl ether,[7] chloroform,[8] acetone,[9] dimethylformamide,[10] dimethyl sulfoxide,[8] ethanol,[11] and methanol.[10] Molar extinction coefficients are plotted against the relative polarity (a normalized empirical parameter of solvent polarity[12]) of the solvent in which they have been measured.

S3. 2DES pulse characterization

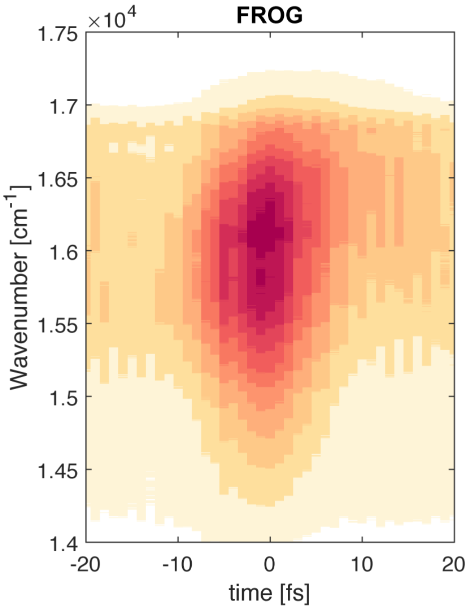

Figure S3. FROG performed in the same experimental conditions of the 2DES experiments.

S4. Additional 2DES data

S4.1 Measures at room temperature

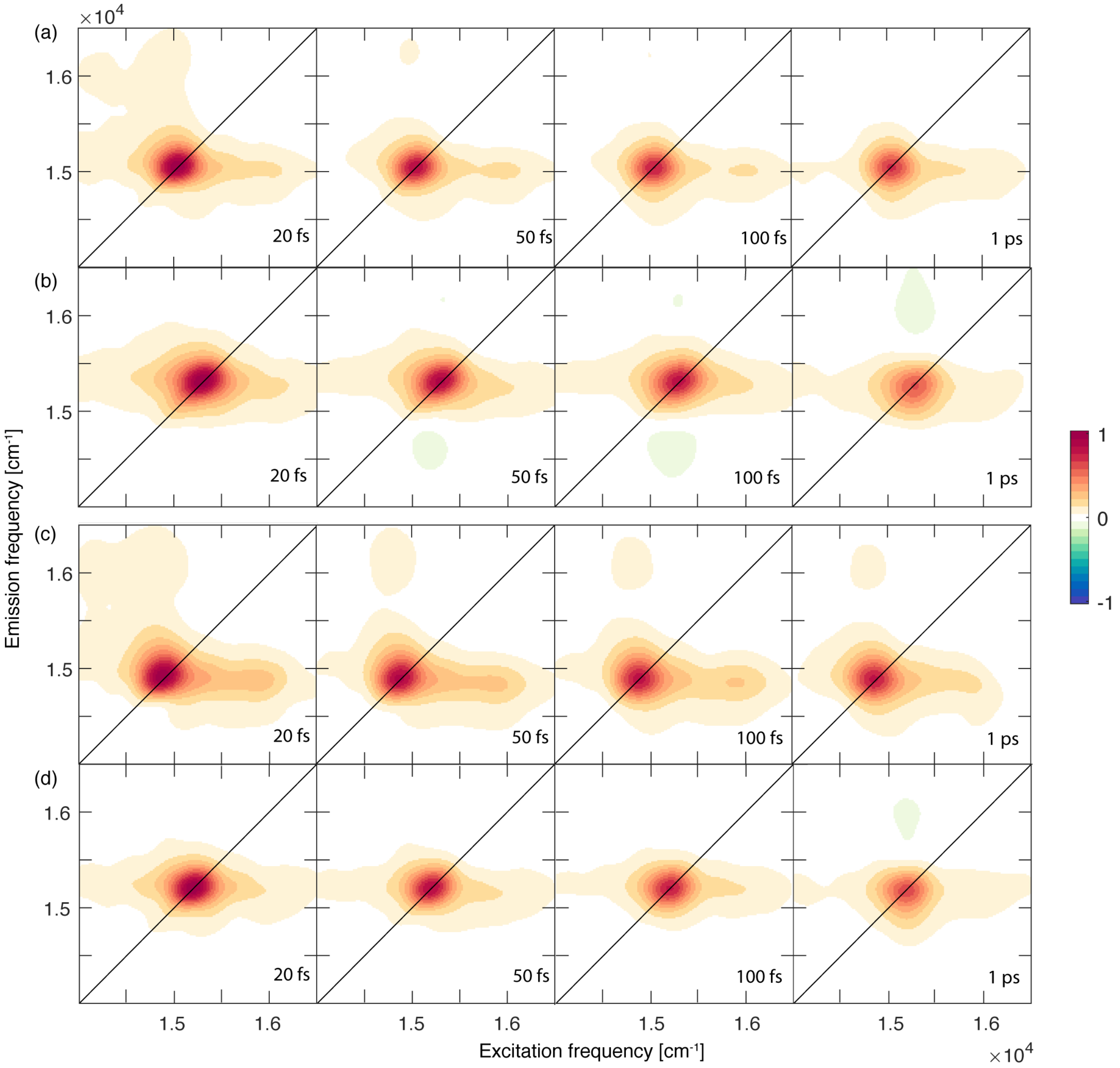

Figure S4. Evolution of purely absorptive 2DES maps at selected values of population time t_2 at room temperature for (a) Lv-*a,* (b) Lv-*b*, (c) Bo-*a* and (d) Bo-*b*.

S4.2 Measures at 77 K

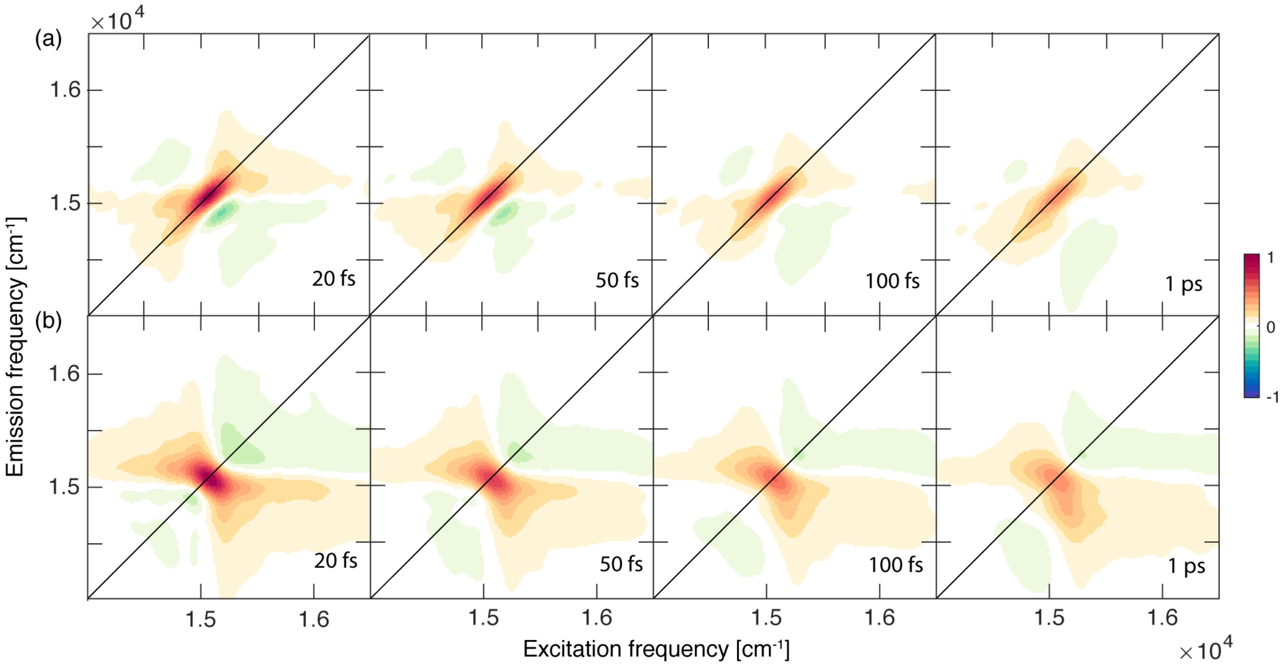

Figure S5. Evolution of (a) rephasing and (b) non-rephasing 2DES maps at selected values of population time t_2 for Lv-*a* at 77 K.

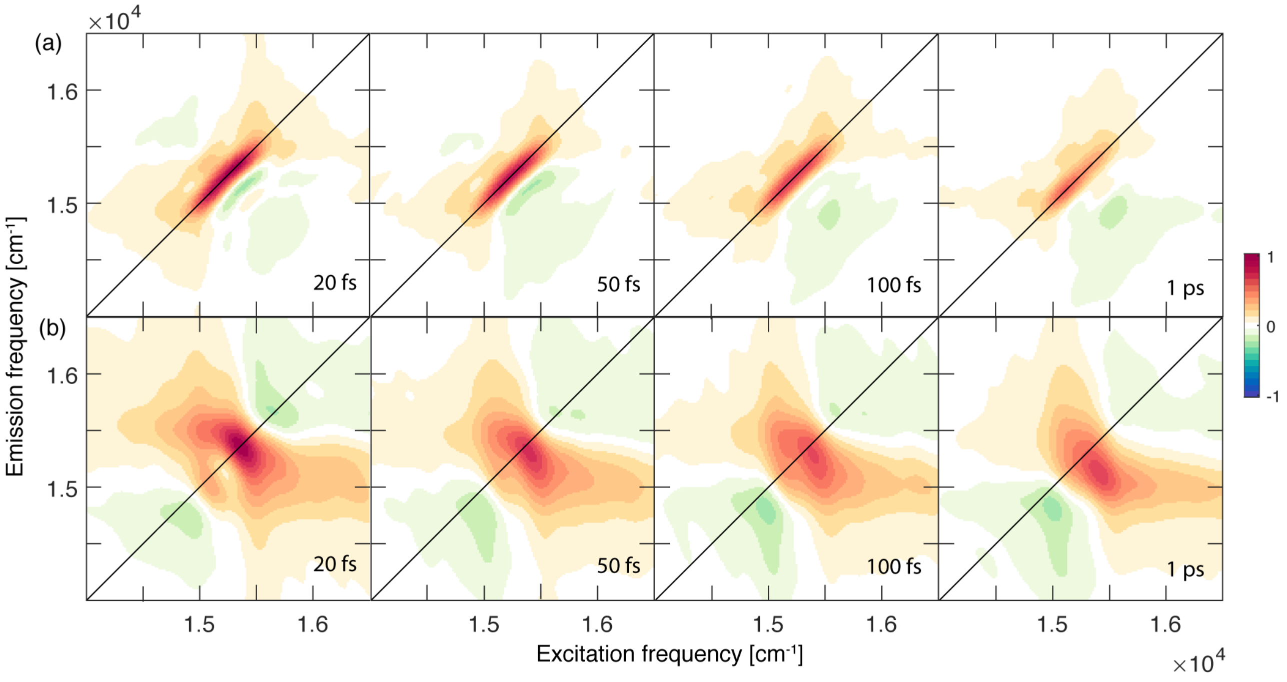

Figure S6. Evolution of (a) rephasing and (b) non-rephasing 2DES maps at selected values of population time t_2 for Lv-*b* at 77 K.

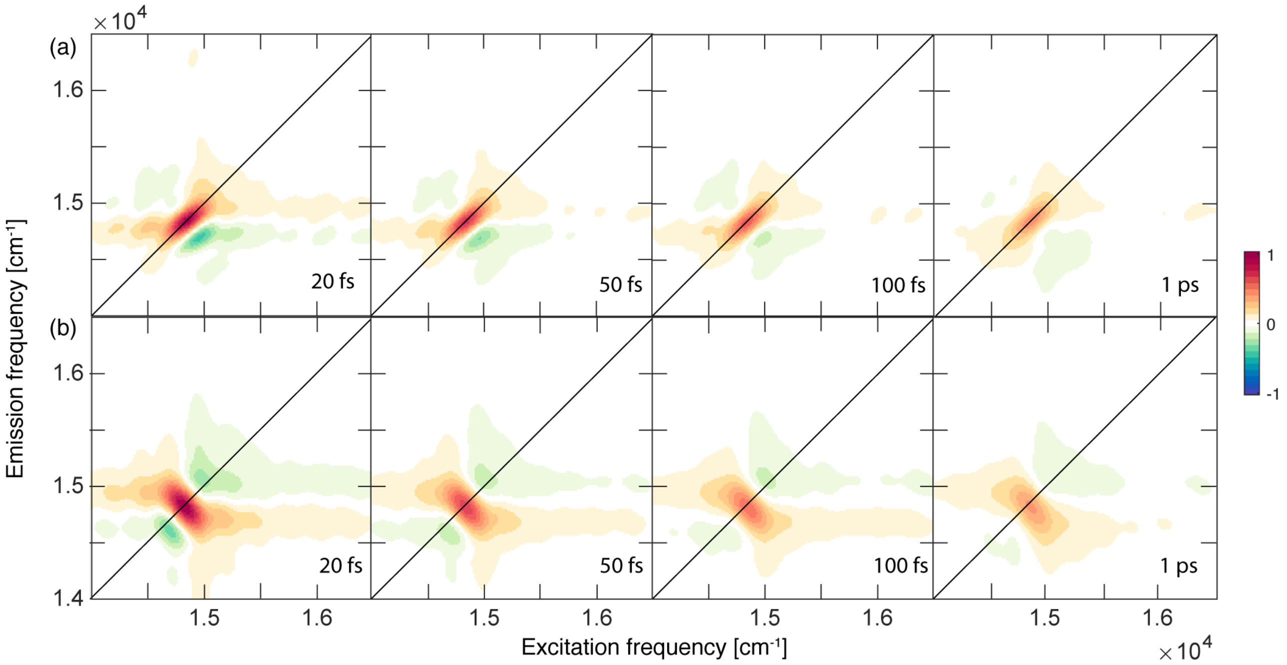

Figure S7. Evolution of (a) rephasing and (b) non-rephasing 2DES maps at selected values of population time t_2 for Bo-*a* at 77 K.

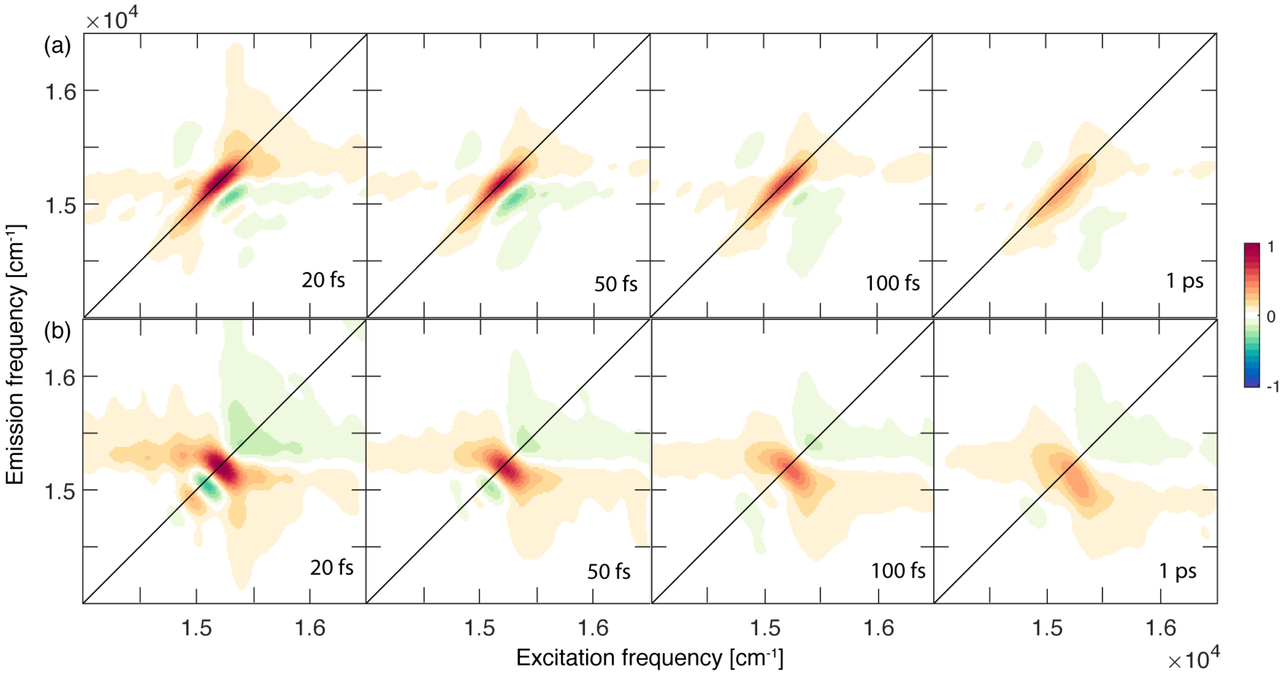

Figure S8. Evolution of (a) rephasing and (b) non-rephasing 2DES maps at selected values of population time t_2 for Bo-*b* at 77 K.

References

(1) Parson, W. W. *Modern Optical Spectroscopy (2nd Ed.)*; Springer-Verlag GmbH: Berlin, 2015.

(2) Knox, R. S.; Spring, B. Q. Dipole Strengths in the Chlorophylls. *Photochem. Photobiol.* **2007**, *77* (5), 497–501. https://doi.org/10.1562/0031-8655(2003)0770497DSITC2.0.CO2.

(3) Horigome, D.; Satoh, H.; Itoh, N.; Mitsunaga, K.; Oonishi, I.; Nakagawa, A.; Uchida, A. Structural Mechanism and Photoprotective Function of Water-Soluble Chlorophyll-Binding Protein. *J. Biol. Chem.* **2007**, *282* (9), 6525–6531. https://doi.org/10.1074/jbc.M609458200.

(4) Lindorfer, D.; Müh, F.; Renger, T. Origin of Non-Conservative Circular Dichroism of the CP29 Antenna Complex of Photosystem II. *Phys. Chem. Chem. Phys.* **2017**, *19*, 7524–7536. https://doi.org/10.1039/C6CP08778G.

(5) Sauer, K.; Smith, J. R. L.; Schultz, A. J. The Dimerization of Chlorophyll a, Chlorophyll b, and Bacteriochlorophyll in Solution 1. *J. Am. Chem. Soc.* **1966**, *88* (12), 2681–2688. https://doi.org/10.1021/ja00964a011.

(6) Watanabe, T.; Hongu, A.; Honda, K.; Nakazato, M.; Konno, M.; Saitoh, S. Preparation of Chlorophylls and Pheophytins by Isocratic Liquid Chromatography. *Anal. Chem.* **1984**, *56* (2), 251–256. https://doi.org/10.1021/ac00266a030.

(7) Hynninen, P. H.; Lötjönen, S. Large-Scale Preparation of Crystalline (10S)-Chlorophylls a and B. *Synthesis (Stuttg).* **1983**, 705–708. https://doi.org/10.1055/s-1983-30476.

(8) Wellburn, A. R. The Spectral Determination of Chlorophylls a and b, as Well as Total Carotenoids, Using Various Solvents with Spectrophotometers of Different Resolution. *J. Plant Physiol.* **1994**, *144* (3), 307–313. https://doi.org/10.1016/S0176-1617(11)81192-2.

(9) Lichtenthaler, H. K. Chlorophylls and Carotenoids: Pigments of Photosynthetic Biomembranes; 1987; pp 350–382. https://doi.org/10.1016/0076-6879(87)48036-1.

(10) Porra, R. J.; Thompson, W. A.; Kriedemann, P. E. Determination of Accurate Extinction Coefficients and Simultaneous Equations for Assaying Chlorophylls a and b Extracted with Four Different Solvents: Verification of the Concentration of Chlorophyll Standards by Atomic Absorption Spectroscopy. *Biochim. Biophys. Acta - Bioenerg.* **1989**, *975* (3), 384–394. https://doi.org/10.1016/S0005-2728(89)80347-0.

(11) Wintermans, J. F. G. M.; De Mots, A. Spectoscophotometric Characteristics of Chlorophylls a and b and Their Pheophytins in Ethanol. *Biochim. Biophys. Acta* **1965**, *109*, 448–453.

(12) Reichardt, C. *Solvents and Solvent Effects in Organic Chemistry*; Wiley, 2003. https://doi.org/10.1002/3527601791.
